# Extended anogenital Buschke-Löwenstein tumor in an immunocompetent patient

**DOI:** 10.11604/pamj.2019.34.108.17999

**Published:** 2019-10-23

**Authors:** Salma Salim, Badreddine Hassam

**Affiliations:** 1 Dermatology and Venereology Department, Ibn Sina University Hospital, Rabat, Morocco

**Keywords:** Buschke-Löwenstein tumor, human papilloma virus, immunocompetent patient

## Image in medicine

We report the case of a 56-year-old man who presented with locally advanced giant condyloma acuminatum (Buschke-Löwenstein tumor) after approximately 15 years of neglect due to hospital phobia. Clinical examination showed an extensive, erosive, exophytic and cauliflower-like growth involving his suprapubic, external genitalia and perianal region. No inguinal or supraclavicular lymphadenopathy was detected clinically. Histological study revealed a papillomatous and acanthotic epithelium with ortho and parakeratosis and koilocyte cells suggestive of HPV infection. No signs of malignant transformation were noted. Radiological investigations consisted of a thoracic-abdominal-pelvic computed tomography scan which showed the localization of this tumor in the external genitalia, perineal and suprapubic region without any lymph nodes or distant metastases. The results of biochemical and serological investigations including HIV test were normal. After discussion among the oncologist, radiotherapist, pathologist and surgeon, the patient initially received chemoradiotherapy, followed by extensive local excision with average outcome. Buschke-Lowenstein tumor is a relatively rare sexually transmitted disease. It is a neoplasm of the anogenital region which has benign appearance on histopathology. It often grows over years in immunocompetent patients and can be highly destructive to local tissue. It carries a high recurrence rate and a significant potential for malignant transformation. Human papilloma virus (types 6 and 11) has been implicated as an etiologic agent for this tumor. Since this disease is rare and no controlled studies exist, radical excision of this anogenital lesion is generally recommended as the first line therapy and close vigilance and follow up are essential.

**Figure 1:**
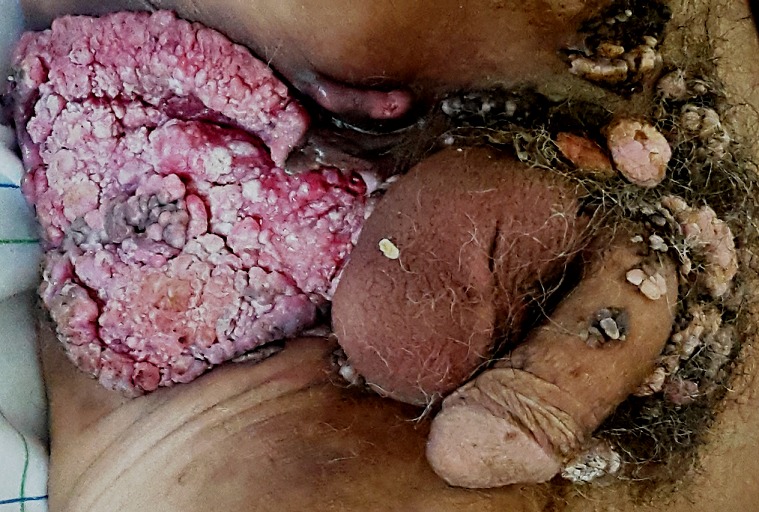
Buschke-Lowenstein tumor presenting as an extensive, erosive, exophytic and cauliflower-like growth involving the suprapubic, external genitalia and perianal region

